# The Hospitals of the Women’s Homœopathic Association of Penna

**Published:** 1888-01

**Authors:** A. J. Frisby

**Affiliations:** Milwaukee, Wis.


### THE HOSPITALS OF THE WOMEN'S HOMŒOPATHIC ASSOCIATION OF PENNSYLVANIA.

Editors Homœopathic Physician:—While I was in Philadelphia for a couple of days during the Constitutional Centennial Celebration, I saw for the first time an article in the June or July number (I forget which), of your journal, intended to reflect discredit upon the homœopathy and general good faith of the women physicians who resigned from the staff of the Women's Homœopathic Hospital in the spring. It was stated in that article that Cod-liver Oil, pills of Ergotin, Morphia, Belladonna, Strychnine, Aloes, etc., had been found in the hospital, and the impression was conveyed that they had been in use there, and that the women physicians were in some way responsible for it.

As I had resigned the position of resident physician in the hospital above mentioned, only a short time before the article appeared, I probably know as much about the matter as anybody. All of the articles mentioned above, together with Pepsine, Bovinine, vin Mariani, etc., were samples left by agents. They were deposited in a corner cupboard in the dispensary, where coal-oil, empty bottles, waste paper, and various kinds of rubbish had accumulated, and where no medicines intended for use in the hospital or dispensary were ever kept. They were there when I took charge of the hospital in the fall, and they were there when I left it in the spring. They were never used while I was in charge of the hospital, and I have no reason to suppose that they were during my predecessor's administration. I cannot understand how any one finding them could fail to perceive that they were sample bottles, or could honestly suppose for a moment that they had been in use in the hospital.

I trust that your sense of justice will move you to publish this statement, thus correcting the very incorrect impression given by the article to which I have reference.

A. J. FRISBY.

MILWAUKEE, WIS., Oct. 24th, 1887.

[We publish the above letter at the request of Dr. Frisby. When we made the statement, to which exception is taken, we were well assured of the truth of our charges. Since the reception of this letter, we have again examined the evidence, and *find no reason to change our original opinion, or to recede from the position we have taken.*—W. M. J.]

